# Non-syndromic hypodontia of maxillary lateral incisors and its association with other dental anomalies

**DOI:** 10.1186/s40510-022-00451-2

**Published:** 2022-12-30

**Authors:** Giana Maria Lupinetti, Peng Li, Kyle Feagin, Mary MacDougall, Ejvis Lamani

**Affiliations:** 1 Department of Orthodontics, School of Dentistry, University of Alabama at Birmingham, 1919, 7th Avenue South, SDB 313, Birmingham, AL 35294-0007 USA; 2 Department of Acute, Chronic and Continuing Care, School of Nursing, University of Alabama at Birmingham, Birmingham, AL USA; 3 Faculty of Dentistry, University of British Columbia, Vancouver, BC Canada

**Keywords:** Tooth agenesis, Maxillary lateral incisor agenesis, Prevalence, Other dental anomalies, Association studies

## Abstract

**Background:**

Tooth agenesis (TA) is the developmental absence of one or more teeth and is the most common craniofacial disorder in humans. Maxillary lateral incisor agenesis (MLIA) is a specific subtype of TA and can have esthetic, functional, and psychosocial implications for patients. The aim of this study was to evaluate the prevalence of MLIA amongst patients with non-syndromic tooth agenesis, as well as its association with other dental anomalies.

**Materials and methods:**

The dental records of 240 patients with non-syndromic congenitally missing teeth treated at the University of Alabama at Birmingham Department of Orthodontics were reviewed. Dolphin Imaging software was used to identify missing teeth, microdonts, peg laterals, impactions, and transpositions. Data were analyzed using chi-square or Fisher’s exact test. All the tests were two-sided at the significance level of 0.05 (SAS 9.4).

**Results:**

In the patient cohort, MLIA prevalence was 37.5% (second most common) and no gender or ethnic differences were identified. We also observed the bilaterally missing lateral incisors more frequently than the unilateral presentation (*p* = 0.0006). Additionally, 62.5% of patients with unilateral MLIA displayed a contralateral tooth that was a peg (*p* = 0.0001); however, no association was found with other microdonts. Furthermore, of the 90 patients missing at least one maxillary lateral incisor, 42.2% were missing another tooth type and 10% of MLIA patients also had an impacted tooth (mainly maxillary canines). However, these were not statistically significant. Finally, no transposed teeth were found in our patients.

**Conclusions:**

This study found that maxillary lateral incisors were the second most frequently missing teeth. When clinicians diagnose congenital absence of a maxillary lateral incisor, the patient should be evaluated for other missing teeth, peg lateral incisors, or potential impactions, especially maxillary canines.

## Background

Tooth agenesis (TA) remains one of the most common human developmental malformations [1, 2]. TA can present as the absence of all teeth, termed anodontia (a very rare condition), or the absence of six or more teeth, called oligodontia. However, the most common form of TA is known as hypodontia, which refers to the failure of fewer than six teeth to develop [3, 4].

Agenesis is seen in both primary and permanent dentitions [3]. However, the incidence of missing permanent teeth is much higher than that of missing primary teeth (2.5–13.4% vs. 0.4–2.4%, respectively) [1, 3, 5–8]. Studies have shown that TA prevalence varies with ethnicity and the geographical distribution with an increase risk in females [1]. A meta-analysis looking at Caucasians from Europe, Australia, and North America showed that in Europeans 4.6% of males and 6.3% of females exhibited hypodontia of the permanent dentition, while Australians had a prevalence of 5.5% and 7.6% in males and females, respectively [1]. This study found a lower frequency of congenitally missing teeth in North American Caucasians (3.2% in males and 4.6% in females).

The etiology of TA is complex and both genetic and environmental factors contribute to its pathology [1]. Agenesis manifests a wide range of expressivity and can occur as an isolated trait or as part of a syndrome [9–14]. The familial form of TA can be transmitted as autosomal-dominant, autosomal-recessive, or X-linked, but could also have an unclear segregation pattern [15]. Additionally, an increasing amount of data shows that a common genetic etiology may predispose individuals to both hypodontia and cancer [13, 16–18]. While mutations at any point in development can result in arrest of tooth formation, agenesis (excluding third molars) is most often seen affecting formation of mandibular second premolars, maxillary lateral incisors, and maxillary second premolars [1, 3, 5, 6].

Maxillary lateral incisor agenesis (MLIA), a subtype of TA with a prevalence of about 2.2%, has critical esthetic, functional, and psychosocial implications for the patients [2, 19]. To clinicians, the congenital absence of one or both maxillary lateral incisors presents a challenge orthodontically and restoratively. While in all other types of hypodontia, unilateral absence of a certain tooth type is more frequent than a bilateral absence, MLIA is observed more as a bilateral condition [2, 19]. Moreover, in all instances of hypodontia both the left side and right side are affected equally except for the maxillary lateral incisor in which the right side is more often affected, while in the primary dentition, the missing maxillary lateral has accounted for roughly 50% of tooth agenesis in Caucasians. In the permanent dentition, values of 0.8 to 4.25% have been reported for the prevalence of missing maxillary lateral incisors in different populations [2, 19].

Several studies involving non-syndromic tooth agenesis have also indicated a possible correlation with other dental anomalies [2, 20, 21]. Cases of MLIA have shown to present with other coronal or radicular malformations, delayed or ectopic eruption of other teeth, impacted teeth, or transpositions. In general, the most frequent tooth abnormality is related to tooth-size and is usually seen in maxillary lateral incisors (excluding third molars). Peg laterals are the most common of these tooth-size discrepancies [22]. Additionally, the tooth often has a shorter root length. The reported overall prevalence of peg laterals has been found to be around 1.8% of the general population but can range anywhere between 0.6 and 9.9% among different populations. It is most common in females and appears more often on the left side. In a 2017 study, Kim et al. found that 19.4% of children presenting with a unilateral peg lateral incisor were missing the contralateral maxillary lateral incisor [22].

In addition to having peg laterals, those with MLIA may have palatally displaced canines, the most frequently impacted teeth aside from third molars [22, 23]. Canine impaction ranges in prevalence from 0.8 to 2.8%, and in 70–85% of the cases it is palatally displaced. Sacerdoti and Baccetti reported significant association of unilateral palatally displaced canines in orthodontic patients with MLIA [24]. Additionally, in a study by Al-Nimiri and Bsoul, 12.6% of subjects having MLIA were found to also have palatally displaced canines [25].

While maxillary canines are commonly impacted, they are also the most frequently transposed teeth [26]. A tooth transposition is defined as a positional interchange of two neighboring teeth. It can also present as the abnormal eruption of a non-neighboring normal tooth. This occurs between the maxillary canine and first premolar or the maxillary lateral incisors and maxillary canines. The average prevalence is around 0.33%, but it can vary from 0.09 to 1.4% among populations [22].

While the prevalence and characteristics of hypodontia have been studied extensively, it was only in the past 10 years that MLIA has started to be identified as a possible subtype of hypodontia. Because of the unique clinical dilemmas and potential of other downstream effects of a mutation MLIA can present, it is imperative to collect more information on the etiology and pathogenesis of this condition. This study examined the prevalence of MLIA amongst patients with non-syndromic tooth agenesis and evaluated its association with other dental anomalies.

## Materials and methods

### Study design and patient sample

**Fig. 1:**
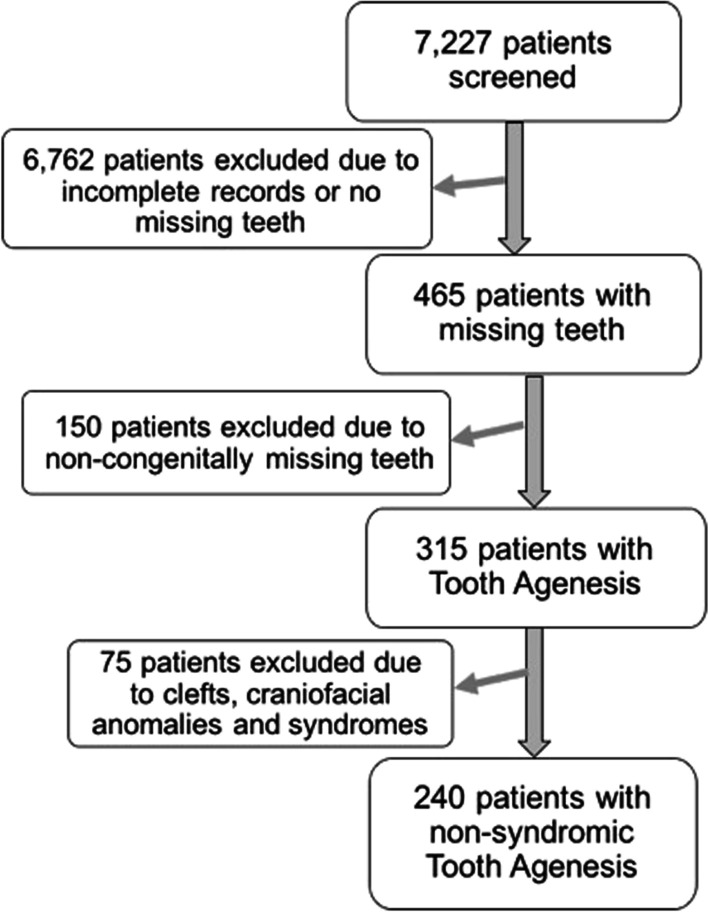
Flow diagram of patients’ selection for the study

Approval for this retrospective study was obtained from the University of Alabama at Birmingham (UAB) Institutional Review Board (IRB#080403015). Dolphin Imaging and Management Solutions Software (Patterson Dental Supply, Chatsworth, CA) was used to search the charts of 7227 patients seen at the UAB Department of Orthodontics between 2000 and 2014 for patients with missing teeth. All patients with missing teeth were identified using panoramic radiographs and initial records (consecutive panoramic radiographs in patients under 12 years of age). We excluded patients with incomplete records (e.g. absent or poor-quality radiographs), patients with non-congenital missing teeth (i.e. teeth extracted due to caries, trauma, previous orthodontics, or other reason), and patients congenitally missing only third molars. We also excluded patients with craniofacial syndromes and/or clefts. This resulted in 240 patients with non-syndromic tooth agenesis (Fig. 1).

### Data collection and variables

From these patients’ charts, we recorded gender, race/ethnicity, as well as age at diagnosis (initial visit). The intraoral photographs and radiographs were used to chart missing teeth by tooth type. The patients’ records were also examined for the presence of other dental anomalies (peg-shaped lateral incisors, transpositions, impactions, and microdonts). A peg-shaped tooth was diagnosed when the incisal mesiodistal width at the crown is smaller than that of the cervical width [22]. A tooth transposition was defined as a positional interchange of two neighboring teeth. It can also present as the abnormal eruption of a non-neighboring normal tooth [26]. Impacted teeth were defined as those unerupted within a normal timeframe and requiring orthodontic intervention or traction to allow eruption into the arch [27, 28].

### Statistical analysis

In this study, we used the alphanumeric notation system for adult teeth and analyzed the data based on the location of the teeth in the arch. The frequency and proportion were used to summarize the outcomes. The group comparisons and association studies were completed using chi-square or Fisher’s exact test where appropriate. All the tests were two-sided at the significance level of 0.05. All analyses were performed using SAS 9.4 (Cary, NC).

## Results

**Fig. 2:**
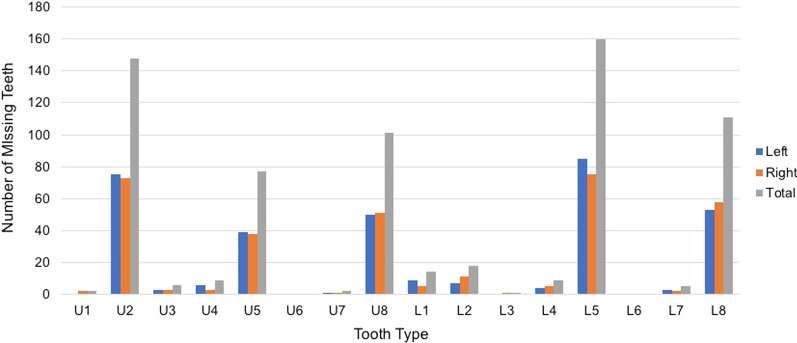
Distribution of missing teeth in non-syndromic tooth agenesis patients

Of the 7227 patients seen at the UAB Department of Orthodontics between 2000 and 2014, we have identified 240 (3.3%) with non-syndromic TA. This sample (ranging in age from 7 to 62 years) consisted of 192 (80.0%) Caucasians, 35 (15.6%) African-Americans, 7 (2.9%) Asians and 6 (2.5%) Hispanics; 103 (42.9%) males and 137 (57.1%) females (Table 1). These patients were missing a total of 451 teeth, of which 148 were maxillary lateral incisors (Fig. 2).

**Table 1:** Characteristics of the non-syndromic tooth agenesis sample

	Female	Male	Both genders
Race/ethnicity			
Caucasian	111 (57.8%)	81 (42.2%)	192 (80%)
African-American	18 (51.4%)	17 (48.6%)	35 (15.6%)
Asian	4 (57.1%)	3 (42.9%)	7 (2.9%)
Hispanic	4 (66.7%)	2 (33.3%)	6 (2.5%)
All races/ethnicities	137 (57.1%)	103 (42.9%)	240
Age (years)			
Mean (SD)	15 (8)	15 (7)	15 (7)
Median (range)	13 (7–62)	14 (7–46)	13 (7–62)

*SD* standard deviation

This study found that maxillary lateral incisor agenesis (U2) was the second most common type of tooth agenesis identified, mandibular second premolar agenesis (L5) being the first (Fig. 2). MLIA was seen in 90 of the 240 (37.5%) patients examined which was statistically lower than non-MLIA (*p* = 0.0001) (Table 2). Of these, 58 patients (24.17%) had bilateral MLIA, while 17 (7.08%) and 15 (6.25%) had unilateral MLIA on the left and right, respectively. There was no significant difference in the prevalence of right versus the left side in cases of unilateral MLIA. However, bilateral MLIA (24.17%) was observed at a significantly higher prevalence than unilateral MLIA (13.33%) (*p* = 0.0006).

**Table 2:** Distribution of the missing incisors in MLIA patients among the non-syndromic tooth agenesis population

MLIA status	N (% in MLIA)	Frequency in the tooth agenesis population	Statistical significance (*p* value)
None	150	62.5%	
Bilateral	58 (64.4%)	24.17%	0.0006*
Unilateral	32 (35.6%)	13.33%	
Left	17 (18.9%)	7.08%	0.7237**
Right	15 (16.7%)	6.25%	
Combined (bilateral + unilateral)	90 (100%)	37.5%	0.0001***

^*^Bilateral compared to unilateral; **comparing unilateral left to right; and ***bilateral + unilateral compared to no MLIA

**Fig. 3:**
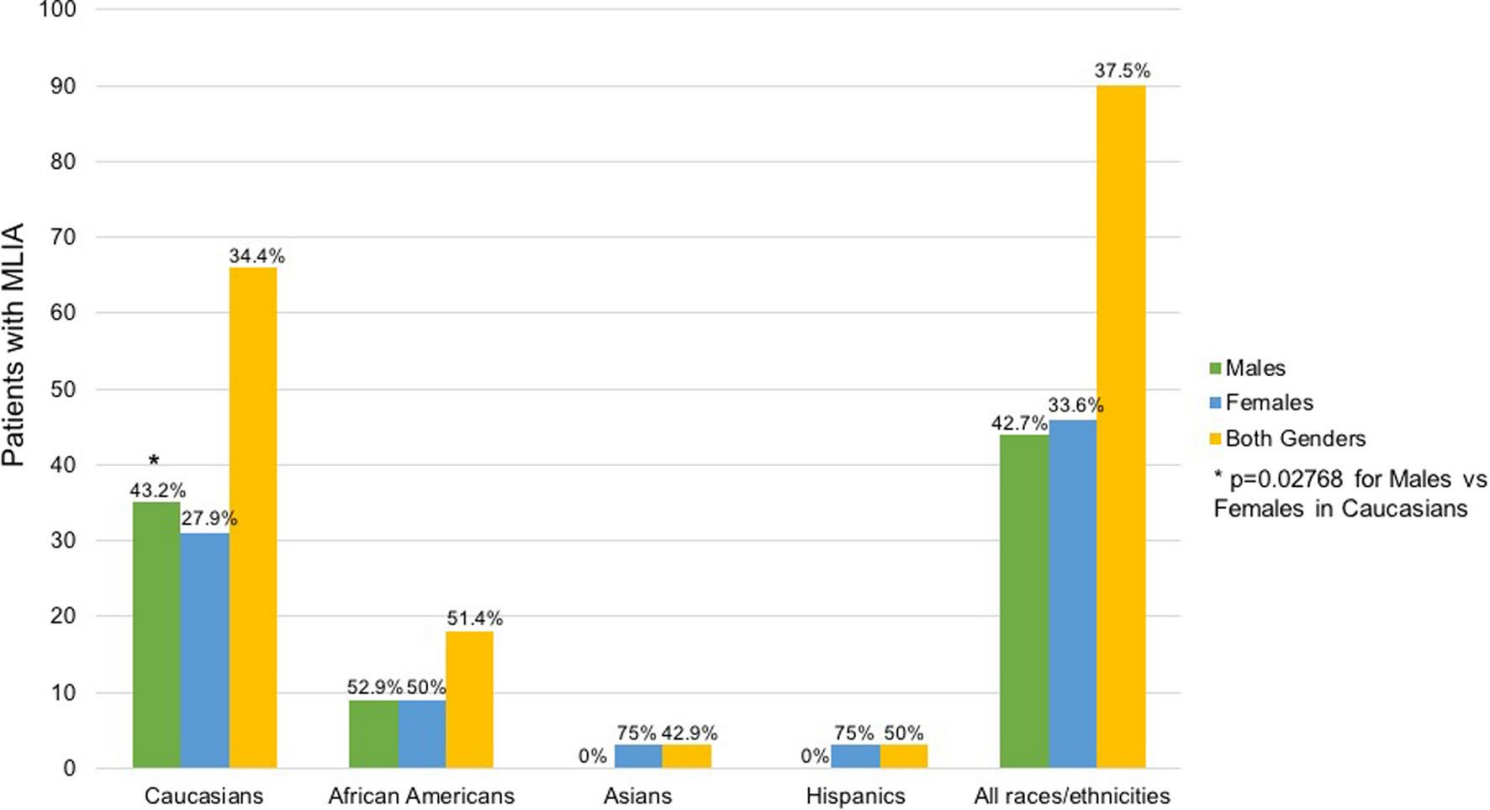
Distribution of MLIA patients by race/ethnicity and gender

MLIA was observed in 34.4% of Caucasians, 51.4% of African-Americans, 42.9% of Asians, and 50% of Hispanics (Fig. 3). However, there were no statistically significant differences in the distribution of MLIA patients by race/ethnicity. Furthermore, while MLIA was seen only in females in the Asian and Hispanic patients, in Caucasians missing maxillary lateral incisors was found significantly more in males than females (*p* = 0.0277). However, overall, there were no statistically significant gender differences in this study (Fig. 3).

Patients with unilateral or bilateral missing maxillary lateral incisors were also evaluated to assess the frequency of another missing tooth (Table 3). We found that 42% of MLIA patients (40% with bilateral and 47% with unilateral) were missing another tooth. This was similar to the frequency of non-MLIA patients having more than one type of missing teeth.

**Table 3:** Association of MLIA with other dental anomalies

MLIA diagnosis	Multiple missing teeth		Peg laterals		Microdonts		Impactions	
	Present	Absent	Present	Absent	Present	Absent	Present	Absent
None	65 (43%)	85 (57%)	18 (12%)	132 (88%)	3 (2%)	147 (98%)	8 (5.3%)	144 (94.7%)
Unilateral	15 (47%)	17 (53%)	20 (62.5%)**	12 (37.5%)	0	32 (100%)	3 (6.3%)	30 (93.7%)
Bilateral	23 (40%)	35 (60%)	0	58 (100%)	0	58 (100%)	7 (12%)	51 (88%)
Combined	38 (42%)	52 (58%)	20 (22%)*	70 (78%)	0	90 (100%)	10 (11%)	80 (89%)

^*^Statistically significant with *p* = 0.0445; **Statistically significant with *p* < 0.0001

The presence of other dental anomalies was also assessed in our non-syndromic congenital tooth agenesis population (Table 3). We found that 40 (15.8%) of these patients present with at least one peg lateral incisor. This prevalence was significantly higher in the MLIA patients than non-MLIA patients (22% vs. 12%; *p* = 0.0445). Furthermore, for the 32 patients with unilateral MLIA, it was more likely to have a remaining lateral that was a peg (62.5% vs. 37.5%; *p* < 0.0001). On the other hand, there was no association between MLIA and the presence of microdonts (*p* = 0.4091). The three patients diagnosed with microdontia of the maxillary second premolars did not present with MLIA (Table 3). In this study, we also examined for tooth transpositions and found that none of our patients presented with transposed teeth.

**Fig. 4:**
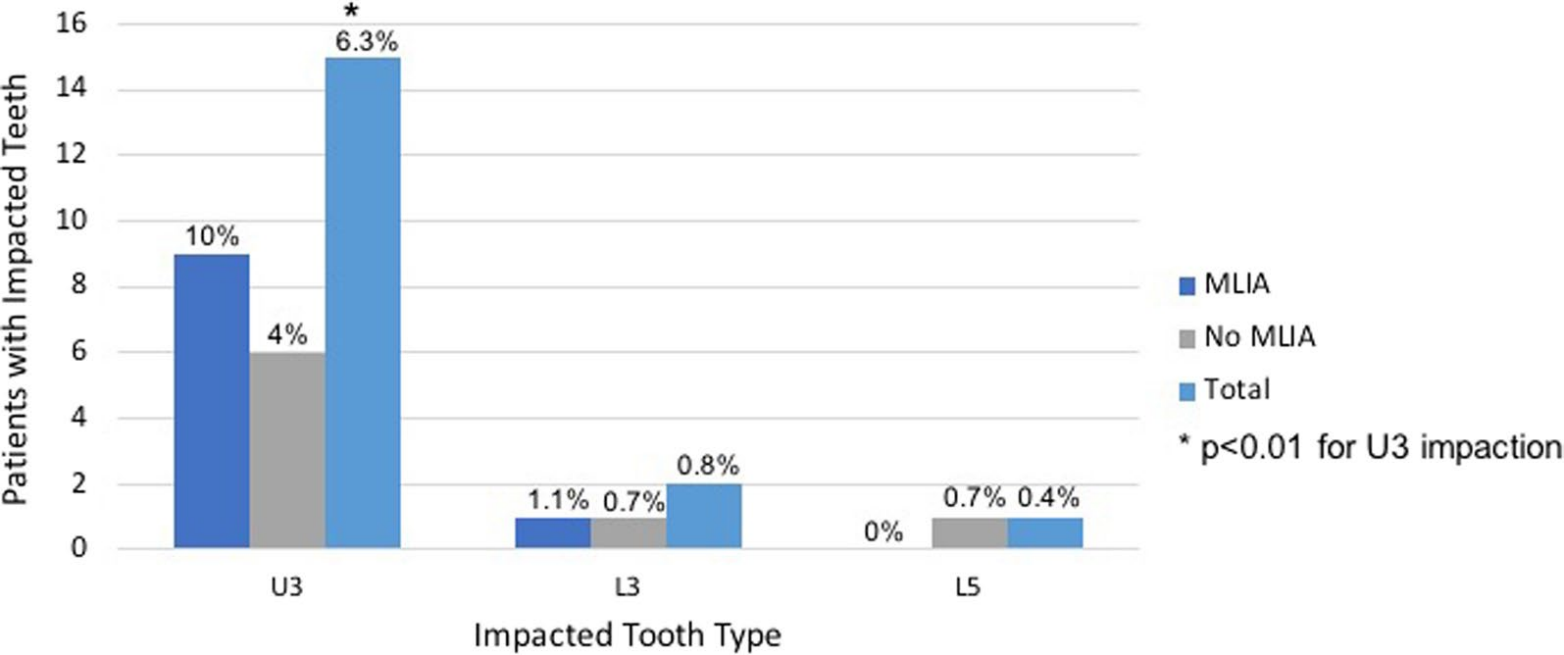
Distribution of impacted teeth among the non-syndromic tooth agenesis patients

Finally, when evaluating the presence of impacted teeth in our sample, we found that among the 18 patients (7.5%) diagnosed with impaction, 83.3% of them presented with impacted maxillary canines. Thus, maxillary canines were the most frequently impacted teeth in our sample (*p* < 0.01) (Fig. 4). However, there was no statistically significant association between MLIA and tooth impaction (*p* = 0.0938) (Table 3).

## Discussion

While hypodontia has been studied in the literature, the prevalence of MLIA is not as well reported. This study provides insight into the characteristics of MLIA and bring awareness to MLIA as a subtype of tooth agenesis. By initially screening a large number of orthodontic patients (7,227 patients), we found that non-syndromic tooth agenesis had a prevalence of 3.3%, which falls toward the lower end of the previously reported range of 2.5–13.4% [1, 3, 5–8]. Furthermore, the 90 patients diagnosed with MLIA in our orthodontic patient sample corresponded to a prevalence of 1.25% for the congenitally missing maxillary lateral incisors. This is slightly higher than what was found in the previous 2017 study by Kabbani et al., which reported a prevalence of 1.15% for MLIA in Syrian children (a non-orthodontic sample) [29]. In addition, similar to the earlier studies, we found that maxillary lateral incisors were the second most commonly congenitally missing teeth (excluding third molars) [1, 3, 5, 6]. This differs from what Vahid-Dastjerdi et al. described in their 2012 study of the Iranian population. Although their MLIA prevalence (35.6%) in the TA patients was similar to ours (37.5%), they reported the maxillary lateral incisors as the most common congenitally missing tooth [30]. In this study, we established that 37.5% of the TA patients had at least one missing maxillary lateral incisor. Furthermore, bilateral MLIA was diagnosed significantly more often than unilateral MLIA (64.4 vs. 35.6%, respectively). This differs from the 2018 study of Brazilian orthodontic patients by Souza-Silva et al., which found that 19.1% of their non-syndromic hypodontia patients were missing only one maxillary lateral incisor and 13.2% presented with bilateral MLIA [23]. However, they did report that the maxillary lateral incisor was the most frequent tooth to be congenitally missing symmetrically.

On the other hand, our study supports the data reported in the meta-analysis by Polder et al. (2004) that found that among patients with affected maxillary laterals, bilateral MLIA occurred more often than unilateral ranging between 50.9 and 57% of the time [5]. They reported that this was unique to MLIA, since in the hypodontia cases involving agenesis of other teeth, unilateral tooth agenesis was more common. Similarly, two other studies of MLIA in Portuguese populations (2005 and 2010) reported bilateral MLIA in 55.2% and 52% of their patients, respectively [19, 31]. Finally, a 2009 study of Brazilian orthodontic patients by Garib et al. found that 51.6% of their MLIA patients presented with bilateral missing lateral incisors [26]. These studies, in addition to ours, indicate that there may be a predilection for bilateral MLIA over unilateral MLIA. These are significant findings, since this phenotype may present particularly challenging consequences phonetically and aesthetically for patients.

In patients with MLIA in order to establish what is considered a normal Angle class I buccal occlusion, space must be opened orthodontically and subsequently restored. This allows maintenance of the canine in its natural position and intercuspation with the first premolar and preserves canine-protected occlusion. However, space opening could be contraindicated in certain patients, for example, those with protruded incisors [2]. Additionally, this commits the patient to a lifetime of maintenance of a fixed or removable restoration at the lateral site. Alternative options such as autotransplantation of a maxillary premolar with incompletely formed roots into the lateral space or complete closure of the lateral space with protraction and subsequent reshaping of the maxillary canines are also frequently employed. Both of these options present difficulties with orthodontic mechanics and aesthetics of replacing a lateral incisor with a larger canine or premolar [2]. Asymmetrical cases (i.e., unilateral MLIA) present even greater aesthetic concerns.

When looking at unilateral presentation of tooth agenesis, the literature review by Rakhshan (2015) reported that hypodontia affects the patient’s left and right sides equally [32]. Our findings support this review as we did not find any significant difference in the prevalence of the left versus right missing lateral incisors (7.08% vs. 6.25%, respectively). However, our data contradict the 2005 study by Pinho et al., which showed that unilateral MLIA on the right was seen more often than on the left (60.3% vs 39.7%, respectively) [31]. This was found to be borderline significant with *p* value of 0.06.

Studies have shown that TA prevalence varies with race, ethnicity, and the geographical distribution of the population. The prevalence of Caucasians diagnosed with TA, summarized by Polder et al. in a 2004 meta-analysis, varied from 3.9% in North America, to 5.5% in Europe, and to 6.3% in Australia [5]. There are less data regarding this disorder in African-Americans. A 2008 study by Harris and Clark reported that 11% of African-Americans had congenitally missing teeth [33]. This was significantly lower than the prevalence of 27% they saw in the Caucasian Americans. However, their study included the absence of third molars in their investigation, which explains their much higher rates compared to the ones reported in the earlier studies. TA rates of Asians also vary by locations and have been reported as 6.9% in Chinese, 9.4% in Japanese, and 11.2% in Korean populations [34–36]. However, these studies do not differentiate between the prevalence of the different types of TA. In our study, we evaluated the racial/ethnic differences in patients with MLIA. We found that while the Caucasians made up the majority (80%) of our sample of patients with congenital tooth agenesis, they had the lowest MLIA prevalence (34.4%). However, this was not statistically significant from the 51.4% in African-Americans, 42.9% in Asians, and 50% in Hispanics. Our data also differ from a recent (2021) study by Eshgian et al., which found that 20.65% of the Hispanics and 6.67% of African-Americans with dental anomalies had congenitally missing teeth [37]. Interestingly, none of their Caucasian or Asian patients presented with TA. Although we were able to investigate racial/ethnic differences in our patient population, the low percentage of Hispanic and Asian TA patients in our sample (2.5% and 2.9%, respectively) presented a limitation for our study.

Previous publications have also indicated that females are more likely to display tooth agenesis. In the 2004 meta-analysis by Polder et al., females were reported to be 1.37 times more likely than males to have non-syndromic tooth agenesis [5]. Another study by Souza-Silva et al. described non-syndromic TA as more common in females (prevalence ratio of 3.49) [23]. Rakhshan’s 2015 review also indicated that females have a greater predilection to non-syndromic hypodontia at a rate of 2:3 when compared with males [32]. Furthermore, the 2005 study by Pinho et al. found the MLIA prevalence to be 1.5% in females and 1.1% in males, which was significant (*p* value < 0.02) [31]. In our study, we also found that non-syndromic TA was more common in females than males (57.1% vs. 42.9%, respectively). In our Asian and Hispanic patients, MLIA was only diagnosed in females. However, unlike Pinho et al., we did not see an increase in risk of MLIA in our overall female population. In contrast, we actually found that Caucasians males present with MLIA statistically more than females. According to our study, overall, females may be more likely to have non-syndromic congenitally missing teeth but not necessarily MLIA.

In this study, we also found that 42% of MLIA patients were diagnosed with agenesis of another tooth type. This is higher than the 2010 study by Garib et al., which showed that 23 of 126 patients (18.2%) had MLIA and another missing tooth [26]. An earlier 2005 study by Pinho et al. also reported that 12.8% of their 219 MLIA patients were missing another tooth [31]. This lower percentage could be explained by the fact that 55 of their 219 patients could not be confirmed for this information and were excluded.

This potential of another congenitally missing tooth is of clinical importance in managing young patients with MLIA, and they should be monitored for other missing teeth. If a young patient present with a missing maxillary lateral, they should be closely monitored for development of the second premolars as they are frequently missing [31]. The restorative dilemma of missing laterals poses a difficult situation as mentioned previously. When other teeth are missing, an even more complex situation develops. Early detection can help in preparing the patients and developing a treatment plan for space closure or long-term restoration. Molecular genetic studies in the future will contribute to identifying the variation in the different patterns of expression of hypodontia.

Furthermore, patients with unilateral MLIA may have an increased risk for the contralateral incisor to be peg-shaped. Hua et al. in a 2013 meta-analysis reported that unilateral MLIA was associated with peg-shaped maxillary laterals 55.5% of the time [38]. Additionally, in the 2010 study by Garib et al., 38.8% of all MLIA patients had peg laterals [26]. They reported an even higher association between unilateral MLIA and peg-shaped contralateral incisor (80.3%). Similarly, in our study we establish a statically significant association between MLIA and peg lateral incisors. We found that 62.5% of unilateral MLIA patients presented with peg laterals. This 62.5% falls within the range reported by Hua et al. and Garib et al. [26, 38]. The literature suggests that the mechanism of agenesis of the lateral incisor is similar to that of the peg shape and that peg laterals are a mild form of hypodontia. The association of peg laterals with unilateral MLIA from our study supports this concept [38].

Several studies involving non-syndromic tooth agenesis have also indicated a possible correlation with other dental anomalies [2, 20, 21]. Cases of MLIA have shown to present with other coronal or radicular malformations, delayed or ectopic eruption of other teeth, impacted teeth, or transpositions. In general, the most frequent tooth abnormality is related to tooth size and is usually seen in maxillary lateral incisors (excluding third molars).

Peg-shaped laterals are not the only anomaly associated with missing lateral incisors. MLIA patients may also be diagnosed with other coronal or radicular malformations, transposition, or impactions [2, 20, 21]. In our study, we identified three patients with microdonts (other than peg laterals); however, none of them presented with MLIA. Furthermore, while tooth transposition may occur in about 0.33% of the general population [22], we did not identify any cases in our patients with non-syndromic TA.

Concerning dental impactions, Sacerdoti and Baccetti have reported a significant association between missing laterals and palatally displaced canines [24]. The maxillary canine is the most frequently impacted tooth after third molars and is followed by the second premolars [25]. In the general population, prevalence of palatally displaced canines ranges between 0.8 and 2.8%; however, Al-Nimiri and Bsoul showed that 12.6% of subjects with MLIA also had palatally impacted canines [25]. In our study, 7.5% of the TA patients also had an impaction. This falls within the range of the prevalence of impacted teeth reported in the general population (5.6–18.8%, excluding third molars) [39]. The overall prevalence in our population is similar to the general population, and there is no difference between MLIA patients and other hypodontia patients. However, unlike Montasser and Taha’s study, which reported that maxillary canines were the second most commonly impacted tooth (after mandibular second premolars) [40], we found that maxillary canines were statistically the most likely tooth to be impacted among all hypodontia patients in our study.

Previous studies have correlated an increase in the prevalence of TA with the Class III skeletal or dental relationship [30, 41]. Celikoglu et al. also found that ectopic eruption was significantly more prevalent in MLIA patients [41]. While these were not variables included in our study, future investigations can further examine the relation between the skeletal and dental classifications and the specific type of tooth agenesis. In addition, these studies can evaluate how this distribution compares to that of the general population.

Due to the complex nature of successful dental development, small changes in eruptive movement can lead to difficulties in tooth movement, function, and esthetics. It is still unclear how these anomalies are genetically related to missing lateral incisors, but it is likely that there are some epigenetic and environmental factors contributing to these conditions and should be studied in the future [32].

## Conclusion

Tooth agenesis remains one of the most common developmental disorders. Specifically, non-syndromic congenital hypodontia of maxillary lateral incisors presents frequently and poses a unique dilemma for dentists and dental specialists both functionally and aesthetically. Understanding the characteristics and demographics of these patients will help in furthering research into the genetic, epigenetic, and environmental factors contributing to the condition.

Our study found that MLIA was the second most common type of TA in patients with non-syndromic congenital tooth agenesis. It occurred more often bilaterally; however, the unilateral presentation was significantly associated with peg-shaped laterals on the contralateral side. This study also found that the frequency of MLIA patients having another missing tooth was greater than previously reported. However, no associations were observed between missing lateral incisors and microdonts or transpositions.

Regarding impacted teeth, although the prevalence of tooth impaction was higher in the MLIA patients, no significant differences were recorded when compared to its prevalence in our non-syndromic TA population. We also showed that maxillary canines were the most frequently impacted teeth. When clinicians diagnose congenital absence of a maxillary lateral incisor, the patient should be evaluated for other missing teeth, peg lateral incisors, or potential impactions, especially maxillary canines.

## Data Availability

The datasets used and/or analyzed during the current study are available from the corresponding author on reasonable request.

## Abbreviations

TA: Tooth agenesis
MLIA: Maxillary lateral incisor agenesis
